# LncRNA Mirt2 Attenuates Osteoarthritis Progression by Promoting miR‐429/TBK1‐Mediated Autophagy

**DOI:** 10.1002/iid3.70423

**Published:** 2026-07-06

**Authors:** Jian‐xin Zhang, Zheng Li, Ping Yang, Kang Pu, Qi Zhou, Hao Yan, Hong‐bo Hu

**Affiliations:** ^1^ Department of Orthopedic Weinan Central hospital Weinan China

**Keywords:** autophagy, LncRNA Mirt2, miR‐429‐5p, osteoarthritis, TBK1

## Abstract

**Object:**

Osteoarthritis (OA) is a chronic and degenerative joint disorder that is prevalent in middle‐aged and older populations. While several long non‐coding RNAs (lncRNAs) have been implicated in OA progression, the role of lncRNA Mirt2 and its regulatory mechanisms remain unclear.

**Methods:**

The expression of lncRNA Mirt2, miR‐429, and TANK‐binding kinase 1 (TBK1) was detected using qRT‐PCR in normal and OA cartilage tissues. The interleukin (IL)−1β‐stimulated chondrocyte was used as an in vitro OA cell. EdU, TUNEL assay, western blot, and ELISA assays were used for our experiments. Luciferase reporter assays, RNA immunoprecipitation (RIP), and RNA pulldown were used to investigate the interactions between lncRNA Mirt2, miR‐429, and TBK1. An *in vivo* OA model was established, and cartilage damage was evaluated using H/E and Safranin O/Fast Green staining.

**Results:**

LncRNA Mirt2 expression was significantly downregulated in OA cartilage tissues and IL‐1β‐stimulated chondrocytes (*p* < 0.05). Transfection of the lncRNA Mirt2 overexpression vector led to a remarkable increase in cell proliferation, a significant reduction in cell apoptosis and inflammation, and a marked elevation in autophagy in IL‐1β‐induced chondrocytes (*p* < 0.05). Functional investigation revealed that lncRNA Mirt2 acts as a competing endogenous RNA (ceRNA) by sponging miR‐429 in IL‐1β‐induced chondrocytes. Additionally, miR‐429 directly targets TBK1. Rescue experiments showed that overexpression of miR‐429 or inhibition of TBK1 effectively counteracted the functional consequences of lncRNA Mirt2 upregulation in IL‐1β‐stimulated chondrocytes, reversing its promotive effects on cell proliferation and autophagy, as well as its inhibitory effect on apoptosis. The *in vivo* experiments indicated overexpression of lncRNA Mirt2 down‐regulated miR‐429, up‐regulated TBK1, substantially attenuated cartilage damage, and accelerated autophagy in OA mice (*p* < 0.05).

**Conclusion:**

LncRNA Mirt2 promoted chondrocyte proliferation and alleviated chondrocyte apoptosis, inflammation, and cartilage degeneration by activating miR‐429/TBK1‐mediated autophagy. Consequently, lncRNA Mirt2 could be a potential target for OA diagnosis and treatment.

## Graphical abstract

LncRNA Mirt2 promoted chondrocyte proliferation and alleviated chondrocyte apoptosis, inflammation, and cartilage degeneration by activating miR‐429/TBK1‐mediated autophagy. Consequently, lncRNA Mirt2 has the potential to be used in the future clinical diagnosis and treatment of OA.

## 1 Introduction

Osteoarthritis (OA), a prevalent degenerative joint condition, exhibits a notably high prevalence among the middle‐aged and elderly demographics [1]. Nowadays, the prevalence rate of OA is increasing year by year due to the aggravation of the aging population in China [2]. The common clinical treatments for OA include drug therapy and joint replacement surgery, which mainly relieve clinical symptoms but cannot cure the disease [3]. Additionally, these traditional treatments do not reduce the incidence of early OA, and patients undergoing joint replacement surgery may also be associated with adverse surgical outcomes and a limited lifespan of the prosthesis [4]. As the current therapy method for OA is limited, it is important to investigate the pathogenesis of OA and its molecular regulation mechanism for the prevention and treatment of the disease.

The onset of osteoarthritis is caused by many factors, including aging, obesity, joint dislocation or injury, and genetic susceptibility [5]. Several studies have shown that osteoarthritis has a strong genetic basis; among these studies, the study of molecular markers is helpful in understanding individual susceptibility and developing new disease therapeutic targets for osteoarthritis [6].

The human genome comprises a mere 2% of protein‐coding RNAs, with the overwhelming majority, approximately 98%, consisting of non‐coding RNAs, including long non‐coding RNAs (lncRNAs) [7]. LncRNAs, which are longer than 200 nucleotides, play regulatory roles in various diseases, including cancer, neurodegenerative, and cartilage diseases [8, 9]. Recent studies have shown that lncRNA Mirt2 expression is abnormally reduced in lipopolysaccharide‐stimulated macrophages, and revealed that lncRNA Mirt2 can act as a negative regulator to affect cellular inflammatory responses [10]. Additionally, lncRNA Mirt2 has been observed to inhibit inflammation in diseases such as nephrosis, colitis, and fatty liver disease [11, 12]. However, the role and effect of lncRNA Mirt2 in OA have not been explored.

The functions of different types of non‐coding RNAs are related, and their interactions can play vital roles in regulating the cellular processes of many diseases such as cancer and inflammatory diseases. MicroRNAs (miRNA) are a type of small non‐coding RNA that regulate the post‐transcriptional expression of mRNA by binding to the 3’‐UTR of the target gene, which leads to translation inhibition or mRNA degradation. The interaction between lncRNA and miRNA can regulate key genes related to subsequent cellular function and disease development. Studies have shown that lnRNA Mirt2 could interact with different miRNAs and influence various diseases [13, 14]. However, the regulatory network underlying the interplay between lncRNA Mirt2 and miRNA in OA remains incompletely elucidated to date.

In our study, we observed down‐regulation of lncRNA Mirt2 in OA cartilage tissues and IL‐1β‐stimulated chondrocytes. Using *in vitro* and *in vivo* models of OA, we further explored the function and underlying mechanism of lncRNA Mirt2. The objective of this study was to elucidate the functional role of lncRNA Mirt2 in OA progression and to investigate its potential regulatory mechanism, focusing on its interaction with specific downstream miRNA and the autophagy pathway. These findings may offer a novel target and theoretical basis for the prevention and treatment of osteoarthritis.

## 2 Materials and Methods

### 2.1 Patients and Specimens

OA cartilage tissues were collected from 30 osteoarthritis patients undergoing knee arthroplasty. The normal cartilage tissues were obtained from 25 patients undergoing traumatic amputations without OA at Weinan Central Hospital. All tissue samples were stored at −80°C before the extraction of RNA. Written informed consent was obtained from all participants, and the study was permitted by the Ethics Committee of Weinan Central Hospital (No. 2023Y009‐1).

### 2.2 Cell Culture and Construction of OA Cell Model

Primary human chondrocytes were isolated from normal cartilage tissues. Briefly, cartilage tissues were first cut into small sections and digested by 0.25% trypsin (Invitrogen, Shanghai, China) and 0.2% collagenase II (Millipore, Boston, MA, USA) for 10 h at 37°C. Next, the cells were filtered using a 200‐mesh filter. Chondrocytes were then obtained and cultured in DMEM/F12 (Hyclone, Salt Lake City, UT) medium supplemented with 10% fetal bovine serum (FBS) and 1% penicillin/streptomycin at 37°C with 5% CO_2_. For IL‐1β stimulation, the third‐generation chondrocytes were treated with 2.5, 5, 10, or 20 ng/mL IL‐1β for 24 h. Then, 10 ng/mL IL‐1β was selected to construct an *in vitro* OA model.

### 2.3 qRT‐PCR

Total RNA was extracted using Trizol reagent, and cDNA was synthesized using the Thermo Revert Aid First Strand cDNA Synthesis Kit (Thermo fisher scientific, Shanghai, China). qPCR was performed using FastStart Essential DNA Green Master on the ABI7300 Real‐Time PCR System. The primers used forq RT‐PCR are: lncRNA miRt2 forward: 5’‐CAT GGG ATC TTG GGA GTC AAT‐3’, reverse: 5’‐CTG GTC TGG AAC TGG AC TT TAC‐3’; miR‐429 forward: 5’‐ CGT CAA CAC TT GC TGG −3’, reverse: 5’‐ CTC AAC TGG TGT CGT GGA −3’; TANK‐binding kinase 1 (TBK1) forward: 5’‐ GAA GAG GAG ACA ACA ACA AGA −3’, reverse: 5’‐GGT AGT CCA TAG GCA TT AG AAG‐3’; GAPDH forward: 5’‐ CTC CCT GGA GAA GAG CTA TGA −3’, reverse: 5’‐ CCA AGA AGG AAG GCT GG AAA −3’; U6 forward: 5’‐ CTC GCT TCG GCA GC ACA −3’, reverse: 5’‐ AAC GCT TCA CGA ATT TG CGT −3’. U6 was used as the internal control for lncRNA or microRNA, and GAPDH was used as the internal control for mRNA.

### 2.4 RNA Fluorescence In Situ Hybridization (Fish)

LncRNA Mirt2 probes, labeled with Alexa Fluor 488, were custom‐designed and synthesized by GenePharma (Shanghai, China). In brief, frozen cartilage tissue sections were hybridized with the Mirt2 probes at 37°C overnight in the dark. After which, the section was incubated with 4’,6‐diamidino‐2‐phenylindole (DAPI) for 30 min. Fluorescence images were captured using a fluorescence microscope (Olympus, IX53, Tokyo, Japan).

### 2.5 Cell Transfection

LncRNA Mirt2 overexpression vector (OE‐Mirt2), control vector (OE‐NC), and small interfering RNA targeting TBK1 (si‐TBK1: 5’‐CCA GAA TCA GAA TTT CTC ATT‐3’) were purchased from Tsingke Biotech (Beijing, China). miR‐429 mimic (5’‐CGU CAA CAC UUG CUG GU UU UCU‐3’) and mimic control (miR‐NC: 5’‐UUC UCC GAA CGU GUC ACG UTT‐3’) were purchased from GenePharma (Shanghai, China). The vectors were separately transfected into chondrocytes 48 h before IL‐1β treatment using X‐tremeGENE 360 transfection reagent (Roche).

### 2.6 5‐Ethynyl‐2’‐Deoxyuridine Assay for Detecting Cell Proliferation

The cells were seeded, 48 h after transfection, into 96‐well plates at 4 × 103 cells per well. After IL‐1β treatment, cell proliferation was measured by a EdU assay. Chondrocytes were incubated with 50 μM EdU solution for 1 h. Fixed with 4% paraformaldehyde, and stained with DAPI for 10 min to mark the nuclei. Finally, EdU‐positive cells were observed under a fluorescence microscope.

### 2.7 TUNEL Assay

After cell transfection and IL‐1β treatment, cell apoptosis was assessed using TUNEL assay. In brief, a TUNEL reaction cocktail containing terminal dexynucleotidyl transferase (TdT; Yeasen Biotechnology, Shanghai, China) and fluorescent labeling solution was added to the cells and incubated for 1 h at 37°C. Next, the cells were stained with DAPI for 5 min.

### 2.8 Western Blot

Total protein was extracted using RIPA buffer, and its concentration was quantified using an amino‐black quantitative detection kit (Jianjiyue Biotech, Xi'an, China). Approximately 20 μg of protein was separated by SDS‐PAGE. The separated bands were electro‐transferred to polyvinylidene difluoride membranes and then blocked with 5% diluted skim milk in Tris‐buffered saline with Tween (TBST) for 1 h. After being rinsed with TBST three times, membranes were incubated with 1:1000 diluted primary antibodies at 4°C overnight, then with corresponding 1:10,000 diluted secondary antibodies for 1 h. Thereafter, the membranes were imaged utilizing an enhanced chemiluminescence (ECL) detection kit (Thermo fisher scientific), and protein signals were analyzed and quantified by the Image J software. Primary antibodies employed in this research includes the following: anti‐Bax (1:1000, ab32503, Abcam, Cambridge, UK), anti‐cleaved caspase‐3 (9662S, Cell Signaling Technology), anti‐Bcl‐2 (Ab196495, Abcam), p‐p38 (Ab195049, Abcam), p38 (Ab170099, Abcam), p‐p65 (Ab76302, Abcam), p65 (ab32536, Abcam), aggrecan (ab313636, Abcam), collagen II (ab307674, Abcam), LC3B (14600‐1‐AP, Proteintech, Shanghai, China), Beclin‐1 (ab207612, Abcam), p62 (5114S, Cell Signaling Technology), TBK1 (28397‐1‐AP, Proteintech), and GAPDH (ab9485, Abcam). The secondary antibodies, goat anti‐mouse IgG antibody and goat anti‐rabbit IgG antibody, were purchased from Proteintech.

### 2.9 ELISA

Levels of TNF‑α, IL‑6, and NO in chondrocyte supernatant or serum were detected by ELISA kits from Elabscience (Wuhan, China).

### 2.10 Immunofluorescence Staining

LC3 punctae in chondrocytes were assessed by immunofluorescence staining. In brief, chondrocytes were first fixed with 4% paraformaldehyde for 15 min, and blocked with 5% bovine serum albumin (BSA) for 1 h. Thereafter, cells were incubated with anti‐LC3B (1:100, Proteintech) at 4°C overnight. Then incubated with FITC‐conjugated goat anti‑rabbit IgG (1:400, Proteintech) for 1 h. Finally, cells were incubated with DAPI for 10 min.

### 2.11 Luciferase Reporter Assay

The wild‐type (WT) and mutant (MUT) sequences of lncRNA Mirt2 or 3’‐UTR of TBK1 were cloned into the psiCHECK™−2 vector (Promega). Chondrocytes were co‐transfected with the recombinant vector with miR‐NC or miR‐429 mimics. After 48 h of transfection, luciferase signals were measured using a dual‐luciferase reporter kit (Promega).

### 2.12 RNA Immunoprecipitation (RIP) and RNA Pulldown Assay

To verify the binding relationship between lncRNA Mirt2 and miR‐429, the RIP and RNA pulldown assays were performed. The RIP assay was carried out using the Magna RIP™ RNA Binding Protein Immunoprecipitation Kit (Millipore, USA). Briefly, chondrocytes were first lysed in RIP lysis buffer. Then incubated with human anti‐Ago2 antibody (ab186733, Abcam) overnight at 4°C. Following this, protein digestion was carried out by incubating each sample with proteinase K. The RNA was purified and quantified by qRT‐PCR. In the RNA pulldown assay, 3’‐biotinylated miR−429 (Bio‐miR‐429) or 3’‐biotinylated miR‐NC (Bio‐NC) was transfected into chondrocytes for 48 h. Next, the cells were harvested and lysed. The samples were then isolated by streptavidin agarose beads, and the expression of lncRNA Mirt2 in pulldown samples was detected using the qRT‐PCR method.

### 2.13 Animal Studies

Animal experiments were approved by the Animal Experimentation Ethics Committee of Xi'an Jiaotong University (No. XJTUAE2023‐2321). A total of 24 BALB/c mice (6–8 weeks of age) were purchased from the Animal Center of Xi'an Jiaotong University (Xi'an, China). Mice were randomly allocated into four groups with six mice per group: control group, in which mice underwent sham surgery; OA group, in which mice underwent destabilization of the medial meniscus (DMM) surgery; OA + LV‐NC group, in which mice underwent DMM surgery and received intra‐articular injection of empty lentivirus (LV‐NC) 1 week post‐surgery; and OA + LV‐Mirt2 group, in which mice underwent DMM surgery and received intra‐articular injection of recombinant lentivirus carrying ncRNA Mirt2 (LV‐Mirt2) 1 week post‐surgery. For OA animal model establishment, DMM surgery was performed to induce osteoarthritis in mice, as previously described [15]. Briefly, after anesthesia, the knee joints were exposed through a medial capsular incision after shaving and disinfecting the surgical area. The medial meniscotibial ligament (MMTL) was transected using microsurgical scissors to destabilize the medial meniscus. In the control group, only the medial capsular incision was made without transecting the MMTL. The joint capsule was then closed and postoperative analgesia was administered via subcutaneous injection of buprenorphine (0.1 mg/kg). One week after surgery, mice in the OA + LV‐NC and OA + LV‐Mirt2 groups received a single intra‐articular injection of 10 μL LV‐NC or LV‐Mirt2 (1 × 10^9^ TU/mL, Obio Technologies, Shanghai, China), respectively. Two weeks after the lentiviral injection, all mice were euthanized by cervical dislocation under deep anesthesia induced by intraperitoneal injection of pentobarbital sodium (80 mg/kg). Blood and cartilage tissues were collected for relevant experimental detection. No mice died or were excluded during the experimental period.

### 2.14 Histological Staining Haematoxylin and Eosin (H/E) and Safranin O/Fast Green Staining

Cartilage tissues obtained from mice were stained with Haematoxylin and Eosin (H/E) and Safranin O/Fast Green. Tissues sections were stained with hematoxylin for 10 min and then eosin for 3 min. For Safranin O/Fast Green staining, tissue sections were first stained with fast green for 5 min and then Safranin O for 30 s. The degree of cartilage damage was evaluated according to the modified Mankin's score and the Osteoarthritis Research Society International (OARSI) scoring system, as described previously [16].

### 2.15 Statistical Analysis

Data are presented as the mean ± standard deviation (SD). Statistical analyzes were performed using GraphPad Prism 9 software. The normality of all datasets was confirmed using the Shapiro‐Wilk test (*p* > 0.05). For the key comparisons involving clinical samples, a post‐hoc power analysis was conducted using G*Power software. This analysis was based on the observed effect sizes (Cohen's d) and the actual sample sizes, with a two‐tailed α level of 0.05. The calculated statistical power exceeded 0.80, indicating sufficient sensitivity to detect the observed effects. For comparisons between two independent groups, the homogeneity of variances was assessed using the F‐test. If variances were assumed equal, an independent samples *t*‐test was applied; otherwise, Welch's corrected *t*‐test was used. For comparisons involving three or more groups, the homogeneity of variances was confirmed using Bartlett's test (*p* > 0.05). One‐way analysis of variance (ANOVA) was subsequently performed, followed by Tukey's post hoc test for all pairwise comparisons. For bivariate correlation analysis, the Pearson correlation coefficient (r) was calculated. A *p* value < 0.05 was considered statistically significant.

## 3 Results

### 3.1 LncRNA Mirt2 Is Down‐Regulated in OA Tissues and IL‐1β‐induced Chondrocytes

**Figure 1:**
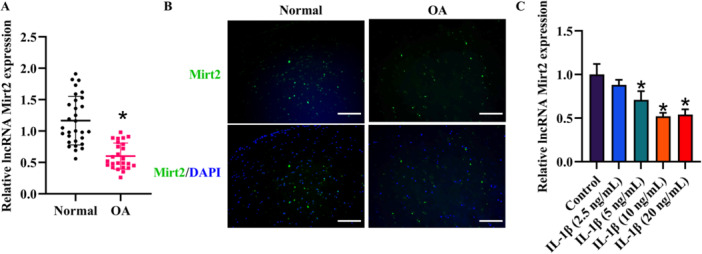
LncRNA Mirt2 is down‐regulated in OA tissues and IL‐1β‐induced chondrocytes. (A) The expression of lncRNA Mirt2 in OA tissues (*n* = 30) and normal tissues (*n* = 25) was detected by qRT‐PCR. *p < 0.05 versus the normal group. (B) Representative images of FISH staining for lncRNA Mirt2 in OA and normal cartilage tissues (x200). Scale bars = 80 μm.(C) The expression of lncRNA Mirt2 in chondrocytes stimulated with different concentrations of IL‐1β (2.5, 5, 10, and 20 ng/mL) measured by qRT‐PCR (*n* = 6 for each group). *p < 0.05 versus the control group.

The qRT‐PCR results showed a remarkable decrease in lncRNA Mirt2 in OA cartilage tissues compared to that in normal cartilage tissues (Cohen's d = 1.8, Figure 1A). This finding was further corroborated by RNA FISH staining, which consistently demonstrated reduced expression of lncRNA Mirt2 in OA cartilage tissues (Figure 1B). To construct an *in vitro* model of OA, chondrocytes were extracted from normal cartilage tissues and stimulated with different concentrations of IL‐1β (2.5, 5, 10, and 20 ng/mL). The data from qRT‐PCR displayed that lncRNA Mirt2 expression was decreased in a dose‐dependent manner after IL‐1β stimulation, and 10 ng/mL IL‐1β treatment had the most significant effect on lncRNA Mirt2 expression (Figure 1C). Thus, 10 ng/mL IL‐1β was used in the following experiment and to construct the *in vitro* OA model. These results revealed that lncRNA Mirt2 was associated with OA.

### 3.2 LncRNA Mirt2 Promotes Proliferation and Inhibits Apoptosis of IL‐1β‐Induced Chondrocytes

**Figure 2:**
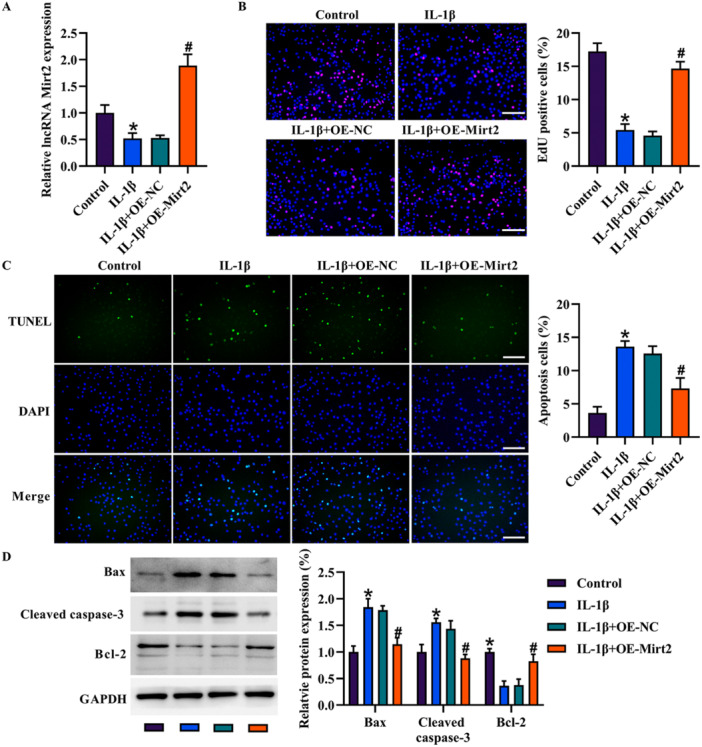
LncRNA Mirt2 promotes proliferation and inhibits apoptosis of IL‐1β‐induced chondrocytes. The control group consisted of normal chondrocyte; the IL‐1β group consisted of chondrocyte treated with 10 ng/mL IL‐1β; the IL‐1β + OE‐NC group consisted of chondrocyte transfected with empty vector and treated with 10 ng/mL IL‐1β; and the IL‐1β + OE‐Mirt2 group consisted of chondrocyte transfected with lncRNA Mirt2 overexpression vector and treated with 10 ng/mL IL‐1β. (A) The expression of lncRNA Mirt2 was measured by qRT‐PCR. (B) Cell proliferation was detected by the EdU assay (x200). Scale bars = 80 μm. (C) Cell apoptosis was measured by a TUNEL assay (x200). Scale bars = 80 μm. (D) Protein expression of Bax, cleaved caspase‐3, and Bcl‐2 was measured by western blot. The values are presented as mean ± standard deviation (*n* = 6 for each group). *p < 0.05 versus control group, #*p* < 0.05 versus IL‐1β group.

To further explore the functional role of lncRNA Mirt2 in OA, we transfected an lncRNA Mirt2 overexpression vector into an IL‐1β‐induced chondrocyte. The results indicated that the expression level of lncRNA Mirt2 was remarkably up‐regulated in the IL‐1β + OE‐Mirt2 group compared to the IL‐1β group (Figure 2A). As shown in Figure 2B, EdU‐positive cells were dramatically declined in the IL‐1β group when compared to the control group. LncRNA Mirt2 overexpression dramatically elevated the proportion of proliferating cells compared to the IL‐1β group. Apart from cell proliferation detected by the EdU assay, cell apoptosis was also measured by the TUNEL assay. Cell apoptosis was dramatically elevated in the IL‐1β group compared to the control group and decreased by lncRNA Mirt2 overexpression (Figure 2C). Moreover, the expressions of pro‐apoptotic proteins Bax and cleaved caspase‐3 were up‐regulated in the IL‐1β group compared to control group and down‐regulated by lncRNA Mirt2 overexpression (Figure 2D). The levels of the anti‐apoptotic protein Bcl‐2 were markedly reduced in the IL‐1β group relative to the control group, but were significantly increased following lncRNA Mirt2 overexpression. These findings showed that overexpression of lncRNA Mirt2 could elevate cell proliferation and restrain cell apoptosis in IL‐1β‐induced chondrocytes.

### 3.3 LncRNA Mirt2 Suppresses the Inflammatory Response of IL‐1β‐induced Chondrocytes

**Figure 3:**
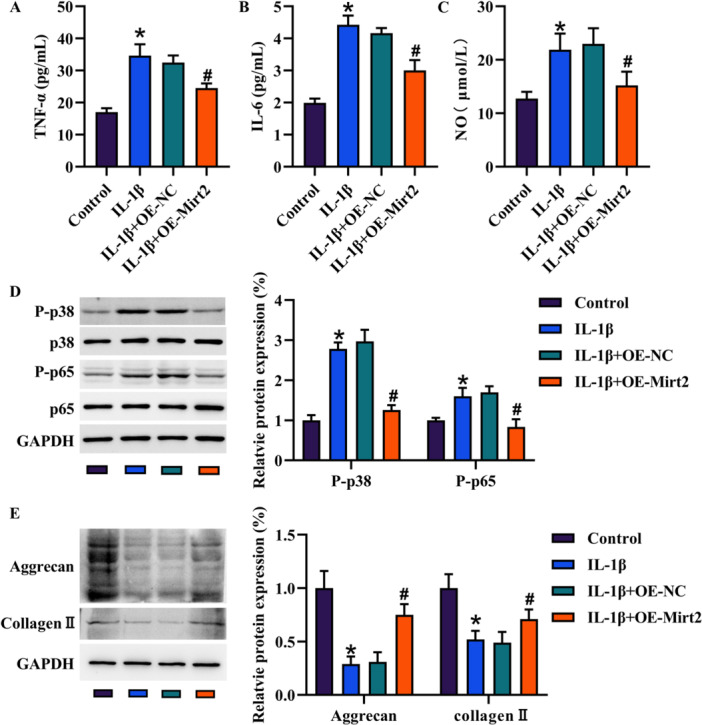
LncRNA Mirt2 suppresses the inflammatory response of IL‐1β‐induced chondrocytes. The levels of TNF‐α (A), IL‐6 (B), and NO (C) were measured by the ELISA method. (D) Protein expressions of p38 and p65 were detected by western blot. (E) Protein expression of aggrecan and collagen Ⅱ detected by western blot. The values are presented as mean ± standard deviation (*n* = 6 for each group). *p < 0.05 versus control group, #*p* < 0.05 versus IL‐1β group.

The ELISA data indicated that IL‐1β treatment significantly promoted, while OE‐Mirt2 transfection inhibited, the levels of TNF‐α (Figure 3A), IL‐6 (Figure 3B), and NO (Figure 3C) in chondrocytes. The activation of p38 MAPK and p65 NFκB proteins is essential in the inflammatory response and is strongly related to the occurrence and progression of OA. As shown in Figure 3D, protein expressions of p‐p38 and p‐p65 were both significantly up‐regulated by IL‐1β treatment and down‐regulated by OE‐Mirt2 transfection. Additionally, lncRNA Mirt2 overexpression significantly restored the expression of aggrecan and collagen II, which were suppressed by IL‐1β stimulation in chondrocytes (Figure 3E). These observations suggested that lncRNA Mirt2 suppressed inflammation in IL‐1β‐induced chondrocytes.

### 3.4 LncRNA Mirt2 Facilitates Cell Autophagy in IL‐1β‐Induced Chondrocytes

**Figure 4:**
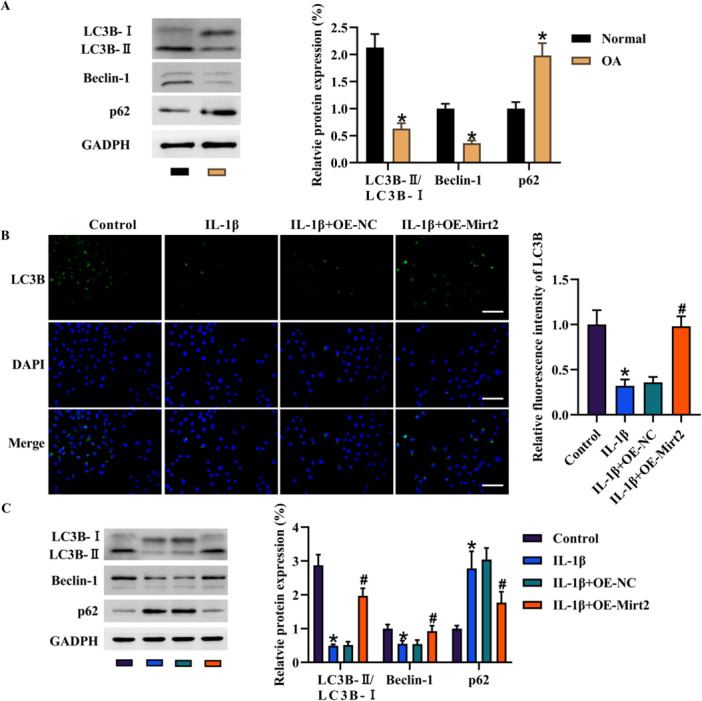
LncRNA Mirt2 facilitates cell autophagy in IL‐1β‐induced chondrocytes. (A) Protein expressions of LC3B‐Ⅰ, LC3B‐Ⅱ, Beclin‐1, and p62 in OA and normal cartilage tissues were measured by western blot. *p < 0.05 versus normal group. (B) Immunofluorescence staining of LC3B in chondrocytes (x400). Scale bars = 40 μm. (C) Protein expressions of LC3B‐Ⅰ, LC3B‐Ⅱ, Beclin‐1, and p62 in chondrocytes detected by western blot. The values are presented as mean ± standard deviation (*n* = 6 for each group). *p < 0.05 versus control group, #*p* < 0.05 versus IL‐1β group.

It has been demonstrated that autophagy plays a crucial regulatory role in inflammation during OA pathogenesis [17]. Analysis of autophagy markers revealed that the LC3B‐Ⅰ to LC3B‐Ⅱ conversion and Beclin‐1 expression were significantly suppressed, while p62 expression was markedly upregulated in OA cartilage tissues compared to normal cartilage tissues (Figure 4A). To explore the potential regulatory role of lncRNA Mirt2 in autophagy, we performed LC3B staining, which showed a significant reduction in autophagy puncta formation in the IL‐1β‐treated group compared to the control group. Notably, lncRNA Mirt2 overexpression effectively increased autophagy puncta formation compared to the IL‐1β‐treated group (Figure 4B). Consistently, LC3B‐Ⅰ to LC3B‐Ⅱ conversion and Beclin‐1 expression were inhibited, and the expression of p62 was boosted by IL‐1β stimulation, whereas transfection of OE‐Mirt2 restored their expression in IL‐1β‐induced chondrocytes (Figure 4C). These findings showed that lncRNA Mirt2 facilitates cell autophagy in IL‐1β‐induced chondrocytes.

### 3.5 LncRNA Mirt2 Functions as a ceRNA by Directly Sponging miR‐429

**Figure 5:**
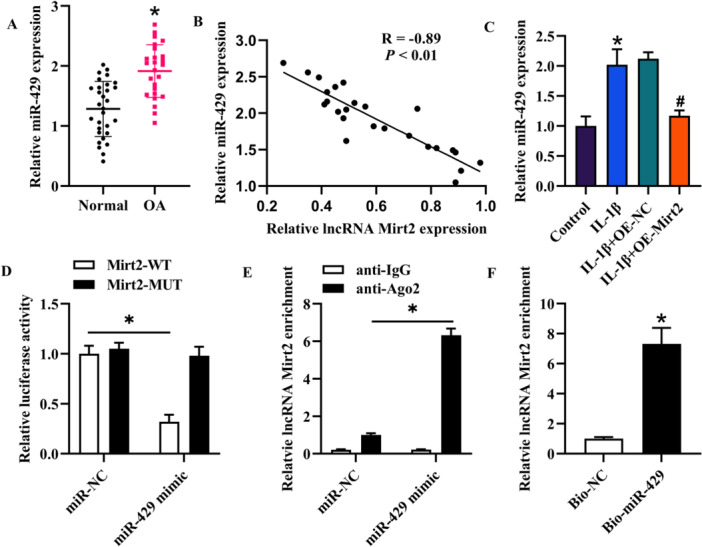
LncRNA Mirt2 functions as a ceRNA by directly sponging miR‐429. (A) The expression of miR‐429 in OA tissues (*n* = 30) and normal tissues (*n* = 25) was detected by qRT‐PCR. *p < 0.05 versus the normal group. (B) Correlation between lncRNA Mirt2 and miR‐429 expression level in OA patients (*n* = 25). (C) The expression of miR‐429 was detected in control, IL‐1β, IL‐1β + OE‐NC, or IL‐1β + OE‐Mirt2‐treated chondrocytes by qRT‐PCR (*n* = 6 for each group). *p < 0.05 versus normal group; #*p* < 0.05 versus IL‐1β group. (D) Chondrocytes were co‐transfected with miR‐NC or miR‐429 mimics and luciferase reporters containing WT or MUT of lncRNA Mirt2. The relative luciferase activity was analyzed 48 h later (*n* = 6). (E) An RIP‐qPCR assay was applied to monitor the enrichment of lncRNA Mirt2 binding to the Ago2 antibody (*n* = 6). (F) The enrichment of lncRNA Mirt2 was measured using qPCR in samples pulled down by biotinylated miR‐429 or Bio‐NC (*n* = 6). *p < 0.05 versus the Bio‐NC group.

A previous study has reported that miR‐429 was a downstream effector of lncRNA Mirt2 in LPS‐induced neuronal cells [18]. In our study, we found that miR‐429 expression was significantly increased in OA cartilage tissues compared to that in normal cartilage tissues (Cohen's d = 1.42, Figure 5A). Pearson's correlation analysis suggested that lncRNA Mirt2 expression was significantly negatively correlated with miR‐429 expression in OA tissues (Figure 5B). Moreover, the expression of miR‐429 was significantly increased in IL‐1β‐induced chondrocytes and decreased with lncRNA Mirt2 overexpression (Figure 5C). The luciferase reporter assay showed that when miR‐429 mimic was co‐transfected with Mirt2‐WT, the luciferase activity was significantly decreased (Figure 5D). Furthermore, the RIP and pulldown assays showed that the endogenous lncRNA Mirt2 was significantly enriched in chondrocytes transfected with miR‐429 mimic or Bio‐miR‐429, revealing the direct binding between lncRNA Mirt2 and miR‐429 and suggesting that lncRNA Mirt2 could function as ceRNA by directly sponging miR‐429 (Figure 5E,F). These data suggested that miR‐429 was a target miRNA of lncRNA Mirt2 in IL‐1β‐induced chondrocytes.

### 3.6 TBK1 Was a Target mRNA of miR‐429 in OA

**Figure 6:**
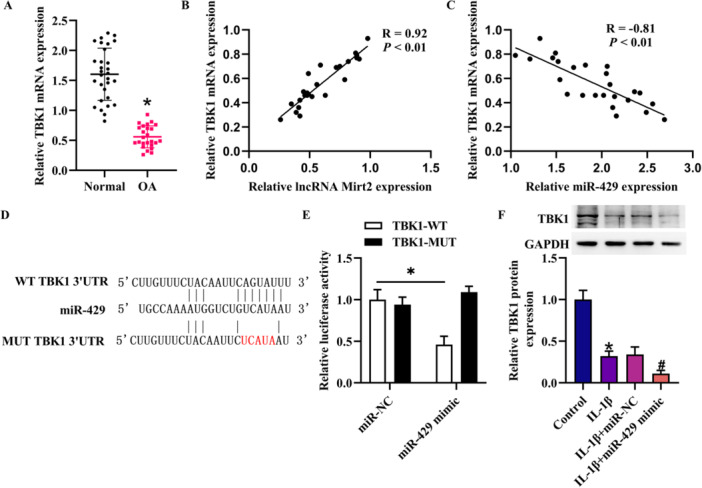
TBK1 was a target mRNA of miR‐429 in OA. (A) The mRNA expression of TBK1 in OA tissues (*n* = 30) and normal tissues (n = 25) was detected by qRT‐PCR. *p < 0.05 versus the normal group. (B) Correlation between TBK1 mRNA and lncRNA Mirt2 expression levels in OA patients (*n* = 25). (C) Correlation between TBK1 mRNA and miR‐429 expression levels in OA patients (*n* = 25). (D) Bioinformatics analysis predicted the putative binding sequences between miR‐429 and WT or MUT 3’‐UTR of TBK1. (E) Chondrocytes were co‐transfected with miR‐NC or miR‐429 mimics and luciferase reporters containing WT or MUT 3’‐UTR of TBK1. The relative luciferase activity was analyzed 48 h later (n = 6). (F) The protein expression of TBK1 was detected in control, IL‐1β, IL‐1β+miR‐NC, or IL‐1β+miR‐429‐mimic treated chondrocytes by qRT‐PCR (*n* = 6 for each group). *p < 0.05 versus normal group; #*p* < 0.05 versus IL‐1β group.

The target gene of miR‐429 was predicted using the online database ENCORI (https://rnasysu.com/encori/index.php). TBK1, among the predicted target genes, has been reported to be closely related to autophagy in OA. As shown in Figure 6A, the mRNA expression of TBK1 was remarkably decreased in OA cartilage tissues compared to that in normal cartilage tissues (Cohen's d = 3.09). Pearson's correlation analysis revealed a positive relationship between lncRNA Mirt2 and TBK1 expression levels (Figure 6B), while a negative correlation was observed between miR‐429 and TBK1 expression in OA patients (Figure 6C). As shown in Figure 6D, the potential target binding site was predicted between miR‐429 and TBK1. The luciferase report assay was applied to verify that miR‐429 directly targeted TBK1 in chondrocytes (Figure 6E). Furthermore, TBK1 protein levels were notably reduced in IL‐1β‐stimulated chondrocytes and were further suppressed following miR‐429 overexpression (Figure 6F). These results implied that TBK1 was a downstream effector of miR‐429 in IL‐1β‐induced chondrocytes, raising the possibility that miR‐429/TBK1 was involved in lncRNA Mirt2‐mediated regulation of autophagy in IL‐1β‐induced chondrocytes.

### 3.7 LncRNA Mirt2 Regulates Cell Proliferation, Apoptosis, and Autophagy of IL‐1β‐induced Chondrocytes via the miR‐429/TBK1 Axis

**Figure 7:**
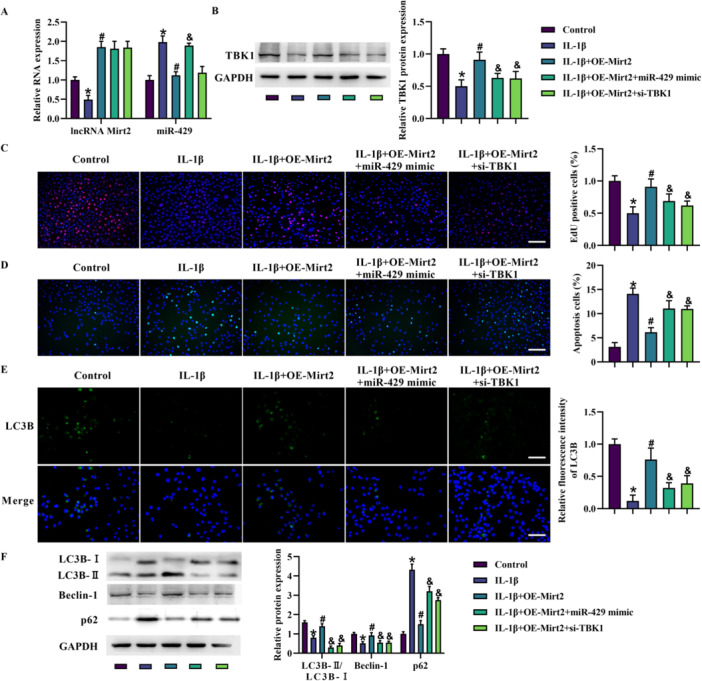
LncRNA Mirt2 regulates cell proliferation, apoptosis, and autophagy of IL‐1β‐induced chondrocytes via the miR‐429/TBK1 axis. The control group consisted of normal chondrocyte; the IL‐1β group consisted of chondrocyte treated with 10 ng/mL IL‐1β; the IL‐1β + OE‐Mirt2 group consisted of chondrocyte transfected with lncRNA Mirt2 overexpression vector and treated with 10 ng/mL IL‐1β; the IL‐1β + OE‐Mirt2+miR‐429 mimic group consisted of chondrocyte co‐transfected with lncRNA Mirt2 overexpression vector and miR‐429 mimic and treated with 10 ng/mL IL‐1β; and the IL‐1β + OE‐Mirt2+si‐TBK1 group consisted of chondrocyte co‐transfected with lncRNA Mirt2 overexpression vector and TBK1 siRNA and treated with 10 ng/mL IL‐1β. (A) The expressions of lncRNA Mirt2 and miR‐429 were measured by qRT‐PCR. (B) Protein expression of TBK1 was measured by western blot. (C) Cell proliferation was detected by the EdU assay (x200). Scale bars = 80 μm. (D) Cell apoptosis was measured by a TUNEL assay (x200). Scale bars = 80 μm. (E) Immunofluorescence staining of LC3B (x400). Scale bars = 40 μm. (F) Protein expressions of LC3B‐Ⅰ, LC3B‐Ⅱ, Beclin‐1, and p62 were measured by western blot. The values are presented as mean ± standard deviation (*n* = 6 for each group). *p < 0.05 versus control group, #*p* < 0.05 versus IL‐1β group, &*p* < 0.05 versus IL‐1β + OE‐Mirt2 group.

To determine whether the lncRNA Mirt2 regulates OA through the miR‐429/TBK1 axis, rescue experiments were performed to test this hypothesis. In the present study, the transfection of miR‐429 mimic significantly increased miR‐429 expression, which was inhibited by lncRNA Mirt2 overexpression in IL‐1β‐induced chondrocytes (Figure 7A). Additionally, the protein expression of TBK1 was induced by OE‐Mirt2 transfection, which was inhibited by knockdown of TBK1 (Figure 7B). Both miR‐429 overexpression and TBK1 inhibition declined cell proliferation, which was, however, promoted by lncRNA Mirt2 overexpression in IL‐1β‐induced chondrocytes (Figure 7C). Cell apoptosis inhibited by lncRNA Mirt2 overexpression was boosted by transfection of miR‐429 mimic or si‐TBK1 (Figure 7D). Furthermore, the promoting effect of lncRNA Mirt2 overexpression on cell autophagy was abolished by miR‐429 mimic or si‐TBK1 (Figure 7E,F). These results suggested that overexpressed lncRNA Mirt2 promoted cell proliferation and autophagy and inhibited cell apoptosis, which was rescued by overexpression of miR‐429 or knockdown of TBK1.

### 3.8 LncRNA Mirt2 Overexpression Attenuated Cartilage Injury in an In Vivo Model of OA

**Figure 8:**
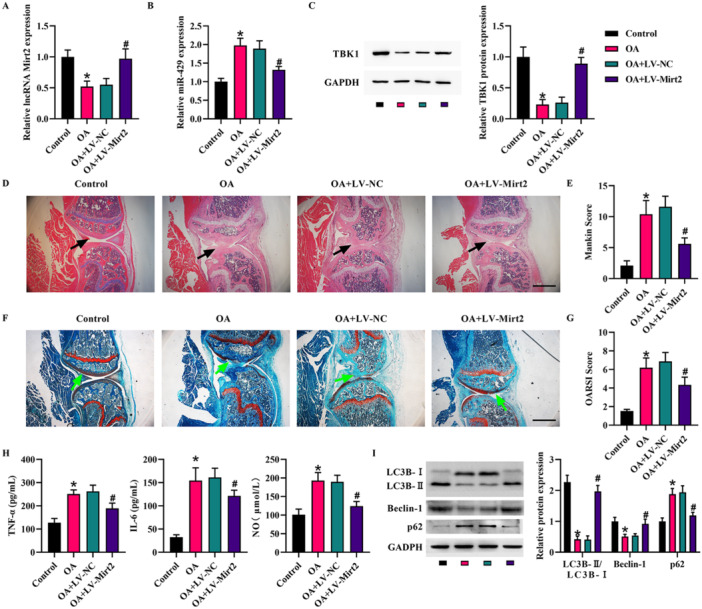
LncRNA Mirt2 overexpression attenuated cartilage injury in an in vivo model of OA. The control group consisted of BALB/c mice underwent sham surgery; the OA group consisted of OA BALB/c mice; the OA + LV‐NC group consisted of OA mice injected with LV‐NC; and the OA + LV‐Mirt2 group consisted of OA mice injected with LV‐Mirt2. The expressions of lncRNA Mirt2 (A) and miR‐429 (B) in cartilage tissues were measured by qRT‐PCR. (C) Protein expression of TBK1 in cartilage tissues was detected by western blot. (D) Haematoxylin and Eosin (H/E) staining of BALB/c mice knee joints (x40). Scale bars = 400 μm. Black arrows point to the articular surface and chondrocyte organization. (E) The modified Mankin's score of the BALB/c mice knee joints. (F) Safranin O/Fast Green (S‐O) staining of mice knee joints (x40). Scale bars = 400 μm. Green arrows indicate areas of proteoglycan staining within the cartilage. (G) OARSI scores of the BALB/c mice knee joints. (H) The levels of TNF‐α, IL‐6, and NO in the serum of BALB/c mice were measured by the ELISA method. (I) Protein expressions of LC3B‐Ⅰ, LC3B‐Ⅱ, Beclin‐1, and p62 in cartilage tissues of BALB/c mice were measured by western blot. The values are presented as mean ± standard deviation (*n* = 6 for each group). *p < 0.05 versus control group, #*p* < 0.05 versus OA group.

To further study the effect of lncRNA Mirt2 in the regulation of inflammation and autophagy *in vivo*, LV‐Mirt2 was used to overexpress lncRNA Mirt2 expression in OA mice. As shown in Figure 8A, lncRNA Mirt2 expression significantly declined in cartilage tissues from OA mice compared to control mice and significantly elevated in the OA + LV‐Mirt2 group. Moreover, lncRNA Mirt2 overexpression inhibited miR‐429 and promoted TBK1 protein expression in OA mice (Figure 8B,C). Histological analysis revealed significant differences between OA and control groups. OA cartilage exhibited surface irregularities, reduced thickness, and disorganized chondrocyte distribution, whereas control cartilage maintained a smooth surface, intact structural integrity, and uniform cellular organization. Safranin O/Fast Green staining further demonstrated severe proteoglycan depletion in OA cartilage, as indicated by markedly diminished red staining intensity, particularly in the superficial zones, in contrast to the strong and homogeneous proteoglycan distribution observed in control specimens (Figure 8D,F). The scores of Mankin and OARSI were dramatically elevated in the OA group compared to the control (Figure 8E,G). LncRNA Mirt2 overexpression alleviated the articular cartilage injury of OA rats and decreased both Mankin and OARSI scores. Additionally, the serum levels of TNF‐α, IL‐6, and NO were significantly boosted in the OA group as compared to the control group and decreased with lncRNA Mirt2 overexpression (Figure 8H). Furthermore, lncRNA Mirt2 overexpression promoted LC3B‐Ⅱ/LC3B‐Ⅰ and Beclin‐1 expression levels while declining p62 levels in OA mice (Figure 8I). These results indicated that lncRNA Mirt2 can alleviate cartilage damage caused by OA via the miR‐429/TBK1 axis in an in vivo model of OA.

## 4 Discussion

Osteoarthritis is characterized by massive cartilage matrix loss, abnormal apoptosis of articular chondrocytes, and chronic inflammation [19]. To date, the pathogenesis of OA remains elusive, hindering the development of efficacious strategies aimed at preventing, mitigating, or even reversing the course of pain in OA patients. Consequently, there is an urgent need to delve deeper into the underlying molecular mechanisms of OA. Earlier research has indicated that lncRNAs play a significant role in OA pathogenesis due to their ability to regulate various cellular processes [20]. LncRNA Mirt2 was reported to be a negative regulator of inflammation in several diseases [21]. Consistent with the previous study, we found that lncRNA Mirt2 expression was significantly reduced in OA cartilage tissues compared to normal tissues and down‐regulated in an *in vitro* IL‐1β‐induced chondrocyte model of OA, suggesting that lncRNA Mirt2 might be involved in regulating OA progression.

Currently, increasing number of studies have demonstrated the important role of lncRNA in regulating cell proliferation in numerous inflammation diseases, including OA [22]. It has been reported that lncRNA Mirt2 ameliorates cell damage in lipopolysaccharide‐evoked PC12 cells by elevating cell proliferation through miR‐429 [18]. In our study, the results confirmed that lncRNA Mirt2 increases cell proliferation of IL‐1β‐induced chondrocytes. Besides upregulating lncRNA, Mirt2 markedly decreased cell apoptosis by suppressing the levels of the pro‐apoptotic protein Bax and cleaved caspase‐3, while concurrently enhancing the expression of anti‐apoptotic protein Bcl‐2.

The anti‐inflammatory capacity of lncRNA Mirt2 has been identified in several diseases [10]. The extent of joint inflammation in OA can be evaluated by measuring inflammatory cytokine levels in both serum and chondrocytes [23]. IL‐1β and TNF‐α are particularly prominent among the proinflammatory cytokines involved in OA progression. IL‐1β is implicated in cartilage destruction and has often been used to construct the in vitro model of OA [24]. TNF‐α seems to be associated with the OA cascade [25]. Apart from IL‐1β and TNF‐α, several other cytokines have also been shown to be closely related to OA. IL‐6 has been reported to reduce proteoglycan content and elevate the production of matrixmetalloproteinase [26]. NO induced by IL‐1β stimulation can trigger the death of chondrocytes [27]. The results of our study showed that in the presence of IL‐1β, lncRNA Mirt2 overexpression inhibited TNF‐α, IL‐6, and NO overproduction. The production of inflammatory cytokines is induced by inflammatory signals via the activation of p38 MAPK and NFκB pathways, which have been reported to contribute to the pathogenesis of osteoarthritis [28]. The data in our study revealed that lncRNA Mirt2 overexpression significantly impeded the phosphorylation levels of p38 and p65, which are known as indicators of the activation of p38 MAPK and NFκB pathways. Furthermore, the expression of essential cartilage matrix proteins, aggrecan and collagen II, was markedly reduced in IL‐1β‐induced chondrocytes, and this reduction was effectively rescued by lncRNA Mirt2 overexpression. These observations indicated that lncRNA Mirt2 protects against OA by inhibiting inflammation.

Autophagy, an intricate intracellular degradation system, orchestrates the phagocytosis and degradation of damaged organelles and biological macromolecules, thereby ensuring metabolic equilibrium and facilitating organelle renewal [29]. This process is particularly vital during cellular stress scenarios, such as starvation, hypoxia, or inflammatory stimulation, autophagy can be activated to prevent cell apoptosis and maintain cellular homeostasis [30]. Existing pieces of research have elucidated a notable suppression of autophagy within the OA microenvironment [31]. Our current study aligns with these findings, demonstrating that the autophagy markers including the conversion of LC3B‐Ⅰ to LC3B‐Ⅱ and Beclin‐1 protein expression were significantly down‐regulated, while p62 expression was up‐regulated in OA cartilage tissues and IL‐1β‐induced chondrocytes. There is increasing evidence showing that lncRNAs exert a vital role in OA by mediating the expression of miRNAs [32]. Our present study found that miR‐429, which is up‐regulated in OA tissues and cells, is negatively regulated by lncRNA Mirt2 in IL‐1β‐induced chondrocytes. Emerging evidence has demonstrated that miR‐429 serves as a critical modulator of autophagy across various pathological conditions [33, 34, 35]. Our experimental results demonstrated that lncRNA Mirt2, functioning as an upstream regulator of miR‐429, effectively rescued IL‐1β‐induced suppression of autophagy in chondrocytes. These findings suggest that lncRNA Mirt2 protects against IL‐1β‐induced chondrocyte damage through activation of autophagy.

TBK1 was predicted and confirmed to be a direct target of miR‐429. TBK1 is a multi‐functional serine/threonine kinase involved in regulating innate immunity, inflammation, and autophagy [36]. It has been reported that TBK1 is down‐regulated in OA patients, and TBK1 overexpression attenuates TNF‐α induced chondrocyte apoptosis by activating autophagy [37]. The data in our study indicated TBK1 was down‐regulated in OA tissues, which is consistent with former research. The rescue experiment showed that lncRNA Mirt2 overexpression increased TBK1 protein expression, which was inhibited by miR‐429 overexpression in IL‐1β‐induced chondrocytes, implying that lncRNA Mirt2 up‐regulated TBK1 expression by inhibiting miR‐429 in the cell progression of OA. Additionally, in our in vitro OA model utilizing IL‐1β‐stimulated chondrocytes, we observed that inhibition of TBK1, similar to miR‐429 overexpression, significantly attenuated the protective effects of lncRNA Mirt2 overexpression. Specifically, both miR‐429 upregulation and TBK1 inhibition reduced cellular proliferation and autophagic activity while enhancing apoptosis compared to lncRNA Mirt2 overexpression alone. These results imply that lncRNA Mirt2 attenuated IL‐1β‐induced chondrocyte damage through the miR‐429/TBK1 axis. The *in vivo* results indicated that lncRNA Mirt2 overexpression decreased miR‐429 expression and promoted TBK1 expression in OA mice, further confirming that lncRNA Mirt2 protects against OA cartilage injury via miR‐429/TKB1.

Despite the promising findings, this study has several limitations that warrant consideration in future research. First, while the therapeutic potential of lncRNA Mirt2 was evaluated in cellular and rodent models, differences in joint anatomy, load‐bearing patterns, and disease progression between rodents and humans necessitate further validation in more clinically relevant large animal models before any translational application. Second, the translation of lncRNA‐based therapies will depend on the development of safe and efficient delivery systems. Future studies should systematically evaluate candidate vectors such as adeno‐associated virus, lentivirus, or engineered extracellular vesicles with regard to their delivery efficiency, stability in the joint environment, and potential off‐target effects. Alternatively, the development of small‐molecule agonizts that enhance endogenous lncRNA Mirt2 expression via high‐throughput screening represents another promising therapeutic strategy.

In summary, the present study verified that up‐regulated lncRNA Mirt2 regulates multiple biological processes in OA, including promoting cell proliferation and autophagy as well as inhibiting cell apoptosis and inflammation by modulating the miR‐429/TBK1 axis. These results provide a novel insight for understanding the pathogenesis of OA, and they indicate that lncRNA Mirt2 may be a promising target for OA treatment.

## Author Contributions

**Jian‐xin Zhang:** conceptualization, data curation, formal analysis, investigation, methodology, writing – original draft. **Zheng Li:** conceptualization, data curation, investigation, methodology, writing – original draft. **Ping Yang:** data curation, investigation, methodology, writing – review and editing. **Kang Pu:** data curation, methodology, writing – review and editing. **Qi Zhou:** methodology, validation, writing – review and editing. **Hao Yan:** methodology, writing – review and editing. **Hong‐bo Hu:** conceptualization, project administration, resources, supervision, writing – review and editing.

## Ethics Statement

Our experiment involved patients tissue samples was approved by the Ethics Committee of Weinan Central Hospital (No. 2023Y009‐1). The animal experiment in this study was supervised by the Animal Experimentation Ethics Committee of Xi'an Jiaotong University (No. XJTUAE2023‐2321).

## Conflicts of Interest

The authors declare no conflicts of interest.

## Data Availability

Data will be made available from the corresponding author on reasonable request.
